# Stent Thrombosis: A Narrative Review From Pathophysiology to Therapy

**DOI:** 10.1002/clc.70286

**Published:** 2026-03-30

**Authors:** Emanuele Cecchi, Andrea Grasso Granchietti, Ruggero Mazzotta, Manuel Garofalo, Giorgia Panichella, Giuseppe Iula, Francesca Maria Di Muro, Silvia Menale, Rossella Marcucci

**Affiliations:** ^1^ Department of Clinical and Experimental Medicine University of Florence, School of Human and Health Science Florence Italy; ^2^ General Cardiology Unit, Department of Cardiac Thoracic and Vascular Medicine Azienda Ospedaliero‐Universitaria Careggi Florence Italy; ^3^ The Zena and Michael A. Wiener Cardiovascular Institute Icahn School of Medicine at Mount Sinai, New York New York USA; ^4^ Cardiovascular Department San Donato Hospital Arezzo Italy; ^5^ Center for Atherothrombotic Disease, Department of Experimental and Clinical Medicine University of Florence Florence Italy

**Keywords:** acute coronary syndrome, drug‐eluting stent, dual‐antiplatelet therapy, hypercoagulability, neoatherosclerosis, stent thrombosis

## Abstract

**Background:**

Stent thrombosis (ST) remains a rare but potentially life‐threatening complication of percutaneous coronary intervention (PCI), associated with high rates of myocardial infarction and mortality. Despite advances in stent technology and antithrombotic therapy, its multifactorial pathophysiology and optimal management remain challenging.

**Methods:**

We performed a narrative review of the current literature focusing on the pathophysiological mechanisms, clinical predictors, and contemporary management strategies of ST. Particular attention was given to stent‐related, pharmacological, and patient‐related factors, as well as emerging therapeutic approaches and preventive strategies.

**Results:**

ST arises from the complex interplay between mechanical factors (e.g., stent malapposition, underexpansion, and neoatherosclerosis), pharmacological aspects (e.g., premature discontinuation or inadequate response to dual antiplatelet therapy), and patient‐related conditions, including hypercoagulability and systemic inflammation. In the acute setting, prompt PCI with restoration of coronary flow remains the cornerstone of treatment, while intravascular imaging plays a key role in identifying underlying mechanisms and optimizing outcomes. Preventive strategies rely on procedural optimization, appropriate antithrombotic therapy, and careful patient selection. New‐generation drug‐eluting stents and imaging‐guided PCI have reduced ST incidence, whereas emerging approaches—including drug‐coated balloons, subcutaneous antiplatelet agents, and anti‐inflammatory therapies—represent promising adjunctive strategies, although ST‐specific benefits are not yet fully established.

**Conclusions:**

ST is a multifactorial complication requiring an integrated approach combining optimized PCI techniques, tailored antithrombotic therapy, and careful risk stratification. Ongoing advances in device technology and pharmacological treatments, along with a growing understanding of inflammatory pathways, may further improve prevention and clinical outcomes.

## Graphical abstract

Stent thrombosis remains a life‐threatening complication of PCI driven by complex interactions between stent design, procedural factors, thrombogenic milieu, and inflammation. This review integrates pathophysiological mechanisms with contemporary and emerging therapeutic strategies, highlighting optimized PCI, tailored antithrombotic therapy, and novel technologies to reduce thrombotic risk.

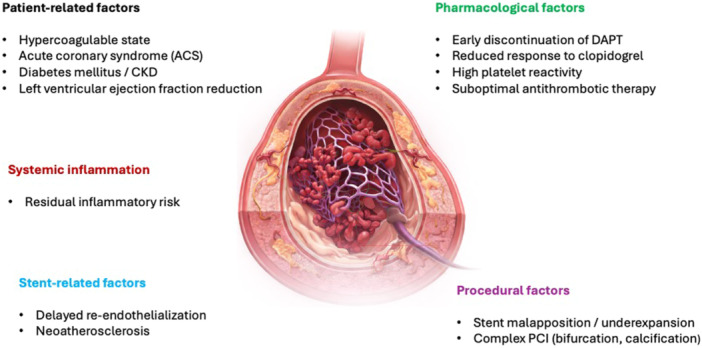

## 1 Introduction

Stent thrombosis (ST) represents a critical complication in patients undergoing percutaneous coronary intervention (PCI), with potentially devastating outcomes, including myocardial infarction (MI) and death. Despite advancements in stent technology and antithrombotic therapy, the risk of ST persists, driven by complex interactions between stent design, mechanical factors, prothrombotic states, and inflammation. Understanding the multifactorial etiology of ST is essential for developing effective prevention and treatment strategies. This review aims to provide a comprehensive overview of the current knowledge regarding the acute management of ST, advancements in stent design, and the role of antithrombotic therapy and inflammation in reducing the incidence of ST. We will also explore the latest clinical evidence and ongoing trials that may shape future practices in the prevention and treatment of this life‐threatening condition.

ST = stent thrombosis;

ARC = Academic Research Consortium; BMS = bare‐metal stent;

DES = drug‐eluting stent; NETs = neutrophil extracellular traps; SES = Sirolimus‐eluting stents;

EES = everolimus‐eluting stents; DAPT = dual‐antiplatelet therapy; OCT = optical coherence tomography; ACS = acute coronary syndrome; ESC = European Society of Cardiology; TF = tissue factor;

STEMI = ST‐segment elevation myocardial infarction; SII = Systemic immune‐inflammation index; hsCRP = high‐sensitivity C‐reactive protein; RIR = residual inflammatory risk;

PCI = Percutaneous coronary intervention;

RA = Rheumatoid arthritis; BVS = bioresorbable vascular scaffold;

MACCE = major adverse cardiovascular events and cerebrovascular; PES = paclitaxel eluting stent.

## 2 Definition and Classification

ST is defined as the thrombotic occlusion of a coronary stent, clinically presenting as a nonfatal MI or sudden cardiac death. The guidelines regarding ST classification were published in 2008 by the Academic Research Consortium (ARC) [1]. Based on the type of stent, we can distinguish ST associated with bare‐metal stent (BMS), first‐generation drug‐eluting stent (DES), and second‐generation DES. Based on timing after initial stent placement, ST can be distinguished in early ST (occurring within 1 month of initial placement), late ST (1−12 months after initial placement), and very late ST (>12 months after initial placement). Early ST is further subdivided into acute (<24 h) and subacute (1−30 days) ST. Definite ST requires an angiographic or pathological confirmation, whereas probable or possible ST are defined clinically [1]. After a careful review of the literature on this topic, we have identified three main potential drivers of ST: stent‐related factors, pharmacological therapy‐related factors, and patient‐ related factors.

## 3 Pathophysiologic Basis of ST

The pathophysiology of thrombosis is historically resumed by Virchow's triad: blood flow stasis, hypercoagulability states, and endothelial alterations. It is well known that platelet aggregation and blood coagulation ultimately result in thrombus formation within the stent, eventually causing its occlusion [2]. Specifically, the stent generates damage to the inner vessel wall, causing endothelial dysfunction or exposure of subendothelial tissue; these events can lead to platelet adhesion, activation, and aggregation. However, the role of inflammatory and immune cells in the pathobiology of ST has not been completely understood so far. Thrombocytes release chemokines that draw immune cells such as monocytes and neutrophils into the developing thrombus; these cells, together with eosinophils, contribute to the release of tissue factor (TF), promote fibrin precipitation, and further enhance platelet activation [3]. A large multicenter European study has been specifically designed to evaluate thrombus specimens in patients with ST [4]. Overall, 253 histopathological thrombus samples derived from a heterogeneous group of patients in terms of both ST timing onset and type of stent were analyzed. The majority of patients presented with late ST (68.8%). Thrombus specimens displayed heterogeneous morphology with platelet‐rich thrombus and fibrin/fibrinogen fragments most abundant. The number of leukocytes was significantly higher as compared to a control group of patients with thrombus aspiration in spontaneous MI. NETs (neutrophil extracellular traps) were observed in 23% of the samples. Eosinophils were present in all types of stents, with higher numbers in patients with late ST in sirolimus‐ and everolimus‐eluting stents (SES and EES, respectively) [4]. These results suggest that leukocytes, particularly neutrophils, seem to be a hallmark of ST, and the presence of NETs supports their pathophysiologic relevance. On the other hand, the presence of eosinophils suggests that there may be an allergic component for certain patients [4]. This had already been hypothesized by the evidence of a higher proportion of eosinophils in durable polymer SES and EES, both with methacrylate as polymer component, as compared with all other stent types [5, 6]. We can summarize factors associated with ST by dividing them into: (1) stent‐related factors; (2) pharmacological therapy‐related factors; (3) patient‐related factors.

### 3.1 Stent‐Related Factors

Several pathogenetic mechanisms have been postulated as possible causes of ST. Since the introduction of BMS into clinical practice in 1986, the initial enthusiasm was largely tempered by the onset of serious early ST episodes. The introduction of DAPT (dual‐antiplatelet therapy) and the improvement in interventional strategies enabled the use of BMS in clinical practice about 10 years later [7]. However, a new issue emerged soon after: neointimal hyperplasia, a complex phenomenon of restenosis characterized by inflammation, smooth muscle cell migration, proliferation, and production of collagen fibers in the extracellular matrix [8]. Neointimal hyperplasia was largely mitigated with the introduction of DESs, which release drugs with antiproliferative action. The most likely and accepted mechanism of ST is the delayed re‐endothelialization due to the antiproliferative effect of the drug released by the stent. This phenomenon facilitates the formation of uncovered struts, an ideal context for thrombus development [6, 8].

Neoatherosclerosis is another contributing factor to ST that has been later confirmed by histopathological and intravascular imaging studies [9]. It is defined as a new plaque generation within the stent, followed by an acute rupture that leads to ST [10]. Foamy macrophage infiltration plays a primary role in neoatherosclerosis. Possible predictors for neoatherosclerosis are younger age, longer implant durations, and underlying unstable plaques [10]. Whether DES is associated with a higher or lower risk of neoatherosclerosis compared with BMS remains a matter of debate. A few optical coherence tomography (OCT) studies have concluded that DES was associated with accelerated neoatherosclerosis compared to BMS [10, 11]. However, the evidence is conflicting, and large RCTs on this topic are lacking.

Stent malapposition is one of the possible mechanisms of ST, especially in highly calcified lesions [12, 13, 14]. Complete and homogeneous stent apposition is particularly challenging in this type of stenosis and can result in incomplete adhesion. An even more common cause of malapposition is the implantation of undersized stents [15]. Coronary angiography is often unable to detect this phenomenon, for which OCT is considered the best diagnostic tool [16].

Histopathologic studies have demonstrated that localized inflammatory and hypersensitivity reactions to some stent components may contribute to ST as well [4, 17]. An allergic reaction in the stent site easily leads to a sort of vasculitis with endothelial dysfunction as the *primum movens* of a process

that will ultimately result in ST [4]. The most likely trigger for a hypersensitive reaction is the polymer matrix. It can indeed occur against any DES component, including the stent metal, the antiproliferative agent, and the durable polymer [4, 17].

### 3.2 Pharmacological Therapy‐Related Factors

The next step was to understand the appropriate duration of DAPT in terms of safety, namely bleeding, and efficacy, that is, the prevention of ST. Indeed, an important mechanism of ST is the early discontinuation of DAPT [18]. Post‐interventional antiplatelet treatment is mandatory either in acute coronary syndrome (ACS) and chronic coronary syndrome (CCS) patients; a default DAPT regimen after PCI is generally recommended for 12 months in ACS [19] and 6 months in CCS [20], unless contraindications are present. In selected patients, the European Society of Cardiology (ESC) guidelines suggest the possibility of a shortened, extended, or in itinere modified DAPT regimen. In CCS ESC guidelines recommend a DAPT regimen that consists mainly of aspirin plus clopidogrel [20].

Nevertheless, about 1/3 of patients show a reduced pharmacological response to clopidogrel with an increased risk for adverse clinical events, including MI and coronary ST [21, 22]; this has led to the definition of residual high platelet reactivity or clopidogrel resistance [23]. More than 10 years ago, two novel P2Y12 inhibitors became available, prasugrel and ticagrelor, and their effects were explored in ACS. In particular, in the TRITON‐TIMI 38 trial (see Table 1), the rate of definite or probable ST was significantly reduced in the prasugrel group as compared with the clopidogrel group, with 68 patients (1.1%) and 142 patients (2.4%), respectively, having at least one occurrence (hazard ratio [HR] 0.48; 95% confidence interval [CI] 0.36−0.64; *p* < 0.001) [24]. Furthermore, an analysis from the prospective, randomized PLATO trial concluded that ticagrelor reduces the incidence of ST as compared to clopidogrel in patients with ACS [25]. The reduction in definite ST was consistent regardless of ACS type, presence of diabetes mellitus, stent type (drug‐eluting or BMS), CYP2C19 genetic status, loading dose of aspirin, dose of clopidogrel before randomization, and use of glycoprotein IIb/IIIa inhibitors at randomization [25, 26]. Results of the aforementioned studies have led recent ESC guidelines on ACS to recommend in these patients a DAPT regimen consisting in aspirin plus ticagrelor or prasugrel, while clopidogrel is recommended only when prasugrel or ticagrelor are not available, cannot be tolerated, or are contraindicated [19]. The rationale of all these recommendations comes from three major randomized control studies, which have been summarized in Table 1 [24, 27, 28].

**Table 1:** Major randomized clinical trials regarding antiplatelet therapy for ACS.

	Study	Population	Randomization	Primary endpoints	Results	Ref.
PCI‐CURE	Multicenter Double‐blind RCT	*n* = 2658 pts with NSTEMI undergoing PCI Mean age 61 years 30% women	Clopidogrel (*n* = 1313) or placebo (*n* = 1345) Patients were pretreated with aspirin and study drug for a median of 6 days before PCI. After PCI, >80% in both groups received thienopyridine for about 4 weeks, after which the study drug was restarted for a mean of 8 months.	Composite of CV death, MI, or urgent target‐vessel revascularization within 30 days of PCI	9 (4.5%) pts in the clopidogrel group had the primary endpoint, compared with 86 (6.4%) in the placebo group (RR 0.70, 95% CI 0.50–0.97, *p* = 0.03). Long‐term administration of clopidogrel after PCI was associated with a lower rate of CV death, MI, or any revascularisation (*p* = 0.03), and of CV or MI (*p* = 0.047).	Mehta et al. [27]
TRITON‐TIMI 38	Multicenter Double‐blind RCT	*n* = 13 608 pts (10 074 with UA or STEMI and 3534 with STEMI) Median age 61 years 26% women	Prasugrel (60‐mg loading dose and 10‐mg daily thereafter) or clopidogrel (300‐mg loading dose and 75‐mg daily thereafter) for 6 to 15 months	*Efficacy endpoint*: death from CV causes, nonfatal MI, or nonfatal stroke. *Safety endpoint*: major bleeding.	The primary efficacy endpoint occurred in 12.1% of pts receiving clopidogrel and 9.9% of pts receiving prasugrel (HR 0.81, 95% CI 0.73‐0.90, *p* < 0.001). ST was less frequent in the prasugrel group (2.4% vs. 1.1%; *p* < 0.001). Life‐threatening bleeding was more frequent with prasugrel (1.4% vs. 0.9%; *p* = 0.01).	Wiviott et al. [24]
PLATO	Multicenter Double‐blind RCT	*n* = 18 624 patients with ACS Mean age 62 years 28% women	Ticagrelor (180‐mg loading dose and 90 mg twice daily thereafter) and clopidogrel (300‐ to 600‐mg loading dose, 75 mg daily thereafter)	Composite of CV death, MI, or stroke	The primary endpoint occurred in 9.8% of pts receiving ticagrelor and 11.7% of those receiving clopidogrel (HR 0.84, 95% CI 0.77−0.92, *p* < 0.001). No significant difference in the rates of major bleeding was found between the ticagrelor and clopidogrel groups (11.6% and 11.2%, respectively; *p* = 0.43).	Wallentin et al. [28]

Abbreviations: ACS, acute coronary syndrome; CI, confidence interval; CV, cardiovascular; HR, hazard ratio; MI, myocardial infarction; NSTEMI, non‐ST‐elevation myocardial infarction; PCI, percutaneous coronary intervention; PCI‐CURE, PCI‐ Clopidogrel in Unstable angina to prevent Recurrent Events trial; PLATO, Platelet Inhibition and Patient Outcomes trial; pts, patients; RCT, randomized clinical trial; RR, relative risk; STEMI, ST‐elevation myocardial infarction; TRITON TIMI‐38, Trial to Assess Improvement in Therapeutic Outcomes by Optimizing Platelet Inhibition with Prasugrel–Thrombolysis in Myocardial Infarction; UA, unstable angina.

### 3.3 Patient‐Related Factors

#### 3.3.1 Clinical Risk Factors

Previous studies have demonstrated the importance of several clinical and instrumental risk factors predisposing to ST [29, 30, 31, 32, 33]. Among them, we can report:

- Premature or improper discontinuation of DAPT is a major and modifiable risk factor, particularly within the first few months after stent implantation;
- History of ST despite adequate antiplatelet therapy;
- ACS at presentation, in fact, patients presenting with ACS, especially STEMI, are at higher risk of ST;
- Reduced left ventricular ejection fraction (LVEF): a low LVEF, indicating reduced heart pumping ability, is associated with increased risk;
- Diabetes mellitus, which is a well‐established risk factor for ST, in particular in the presence of diffuse atherosclerotic lesions;
- Renal insufficiency, a condition that can increase the risk of clotting and thrombosis;
- Advanced age: older patients may have a higher risk, potentially due to age‐related decline in vascular health;
- Smoking that is a known risk factor for cardiovascular disease and can contribute to ST;
- Prior to PCI, in fact, patients with a history of prior stent placement may be at increased risk;
- Use of first‐generation DES and complex PCI procedures.

## 4 Hypercoagulability

Finally, one of the components of Virchow's triad is hypercoagulability. In the setting of ST, inherited thrombophilia disorders can play a key role. In a study by Zavalloni et al., a specific association between studied gene variations and ST has not been detected [34]. Among 127 patients with documented ST, the prevalence of G1691A Factor V Leiden mutation, G20210A Factor II mutation, and C677T MTHFR homozygous polymorphism did not differ significantly among patients with or without ST. Moreover, no association between gene variations and ST was found (odds ratio [OR] 0.61; 95% CI 0.24–1.60; *p* = 0.32) [34]. A case‐control study by Loeffen et al. has evaluated the prevalence of a hypercoagulable profile in patients with ST [35]. Sixty‐three patients after stent implantation were included, of which 23 with ST and 40 without ST. The ST group showed a hypercoagulable state in terms of thrombin generation measured using TF triggers. Active site‐inhibited factor VIIa and recombinant thrombomodulin were added to study the contact activation system and the protein C pathway, respectively. The Authors concluded that this hypercoagulable profile was explained by enhanced contact activation and attenuation of anticoagulation by the protein C pathway [35]. On the other hand, studies assessing the true incidence of ST in patients with acquired thrombophilia (e.g., antiphospholipid antibody syndrome) or chronic myeloproliferative disorders, such as polycythemia vera and essential thrombocythemia, are lacking. The only available evidence comes from a few case reports, primarily on antiphospholipid antibody syndrome, which seem to suggest an association between these acquired thrombophilia states and an increased risk of ST [36, 37, 38, 39, 40, 41].

## 5 Inflammation

Although anti‐inflammatory therapies have consistently demonstrated reductions in major adverse cardiovascular events (MACE) in patients with ACS and CCS, direct evidence supporting their efficacy in preventing ST in humans is currently lacking. Cytokine storm‐related hypercoagulability may play a role in the pathogenesis of ST. Some evidence comes from studies on patients with SARS‐CoV2 infection. Moreover, COVID‐19 is indeed characterized by markedly elevated systemic levels of inflammatory cytokines (IL‐6 and TNF‐α), fibrinogen, and D‐dimer. Several studies have demonstrated higher rates of ST and hospital mortality in patients with COVID‐19 who were stented for ST‐segment elevation MI (STEMI) compared to patients with STEMI without COVID‐19 [42, 43, 44]. Cornelissen et al. have specifically evaluated the effects of a simulated COVID‐19 cytokine storm on stent thrombogenicity. They have observed that, under simulated cytokine storm conditions, fluoropolymer‐coated stents showed the most favorable anti‐thrombogenic and anti‐inflammatory properties [45].

Systemic inflammation, common to several different pathological conditions, may represent an underlying substrate promoting ST. The systemic immune‐inflammation index (SII) is a quantitative measurement of the systemic immune‐inflammatory response. It is defined as follows: SII = *P × N/L*, where P, N, and L are peripheral platelet, neutrophil, and lymphocyte counts, respectively [46]. A recent retrospective study has specifically evaluated the association of the SII with ST in 887 MI patients after stent implantation [46]. The SII ≥ 636 and the number of stents ≥ 4 were found to be independent risk factors for ST after coronary stent implantation [47].

Nearly 60% of ACS patients have a high level of high‐sensitivity C‐reactive protein (hsCRP), a biomarker of systemic inflammation and a predictive factor of high cardiovascular mortality‐defined

as the residual inflammatory risk (RIR) in patients suffering coronary syndromes [48]. Additionally, in a recent analysis of 11 327 patients who underwent PCI, it has been shown that elevated laboratory markers of acute inflammation were associated with the occurrence of ST in both patients with ACS (HR, 2.63; *p* < 0.001) and in patients with stable ischemic heart disease (HR, 3.57; *p* < 0.001) [49]. Several lines of evidence suggest that RIR persists in ACS patients despite guideline‐directed intensive lipid‐lowering therapy and lifestyle modification [50, 51]. Therefore, the pathogenesis and management of ACS should also focus on reducing RIR beyond antiplatelet and lipid‐lowering therapy in the future. Usually, at the level of the ulcerated plaque, activated macrophages and T cells release cytokines responsible for a vicious circle that further increases the thrombotic burden [52]. Therefore, systemic and local inflammatory responses are important causes of ACS, and in fact, systemic inflammatory diseases are associated with an increased susceptibility for acute cardiovascular events: for example, it has been demonstrated that rheumatoid arthritis (RA) is associated with a higher incidence of premature cardiovascular events and a twofold increase in the incidence of ACS [53]. Additionally, RA patients who respond well to anti‐inflammatory medication have a decreased risk of future ACS [54].

## 6 Treatment

We can resume the treatment of intrastent thrombosis into two stages: acute management and preventive strategies.

## 7 Acute Management

ST represents a challenging scenario, with high rates of morbidity and mortality, often leading to nonfatal MI or cardiac death, reaching an incidence of 15%−30% within 30 days of the event [55].

**Figure 1:**
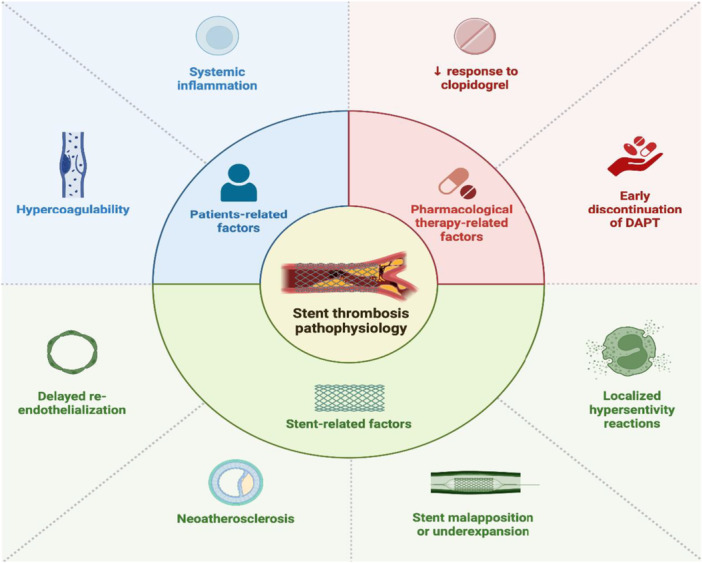
Pathophysiology of stent thrombosis. DAPT, dual antiplatelet therapy.

Most patients with ST are admitted with a STEMI or a NSTEMI diagnosis: in the acute setting, the indicated treatment is the primary PCI [56, 57], and restoration of TIMI flow 3 is mandatory for a favorable patient prognosis. The presence of a significant thrombotic burden can make revascularization complex and increase the risk of distal embolization or the need for additional stent implantation [58]. For intrastent thrombosis revascularization, the use of thrombus aspiration devices (mechanical or rheolytic) remains a matter of debate. Several multicenter randomized trials have been conducted to evaluate the efficacy of these techniques. In the *Thrombus Aspiration in ST‐Elevation MI in Scandinavia* (TASTE) trial—a multicenter, prospective, randomized study—the use of manual thromboaspiration did not improve prognosis or the incidence of reinfarction compared with the group that did not undergo thromboaspiration [59]: the results of the trial, in fact, show that death from any cause occurred in 2.8% of the patients in the thrombus‐aspiration group (103 of 3621), as compared with 3.0% in the PCI‐only group (110 of 3623) (HR, 0.94; 95% CI, 0.72 to 1.22; *p* = 0.63); incidence of hospitalization for recurrent MI at 30 days were 0.5% and 0.9% in the two groups, respectively (HR, 0.61; 95% CI, 0.34 to 1.07; *p* = 0.09), and the rates of ST were 0.2% and 0.5%, respectively (HR, 0.47; 95% CI, 0.20 to 1.02; *p* = 0.06). In addition, thromboaspiration is not a procedure without complications, in case the thrombus is not completely aspirated from the guiding catheter and can then embolize to other organs, with increased risk of ischemic stroke [60, 61]. These results were achieved by an additional multicenter randomized trial (TOTAL trial), in which it was shown that, in patients with high thrombotic burden, the use of manual thromboaspiration did not reduce mortality at 30 days (HR, 0.78; 95% CI: 0.61 to 1.01; *p* = 0.06) and at 1 year (HR, 0.88; 95% CI: 0.72 to 1.09; *p* = 0.25), increasing the risk of stroke (31 [0.7%] hrombus aspiration vs. 16 [0.4%] PCI alone, HR: 1.90; 95% CI: 1.04 to 3.48, *p* = 0.03) [62]. Based on these data, even the ESC Guidelines 2023 for the management of ACS did not recommend the routine use of thromboaspiration, recommending it only in the case of significant residual thrombotic burden after recanalization of the vessel with a guide wire or a balloon [19] (Figure 1).

The hypothesis of reducing the risk of systemic embolization by the use of pharmacological instead of mechanical thrombus destruction was evaluated by the INFUSE‐AMI trial *(Intracoronary Abciximab Infusion and Aspiration Thrombectomy in Patients Undergoing PCI for Anterior STEMI*): in the group assigned to the intracoronary bolus of the glycoprotein IIb/IIIa inhibitor abciximab, the infarct size ‐assessed by cardiac MRI‐ was significantly lower than in the control arm (15.1 vs. 17.9, *p* = 0.03); however, no differences regarding other clinical endpoints, namely MACE (6.6% vs. 7.2%, *p* = 0.81), death (3.1% vs. 2.7%, *p* = 0.81), reinfarction (0.5% vs. 0.9%, *p* = 0.55), ST (1.4% vs. 0.5%, *p* = 0.33), and TIMI major bleeding (1.3% vs. 2.8%, *p* = 0.30) were shown [63].

**Figure 2:**
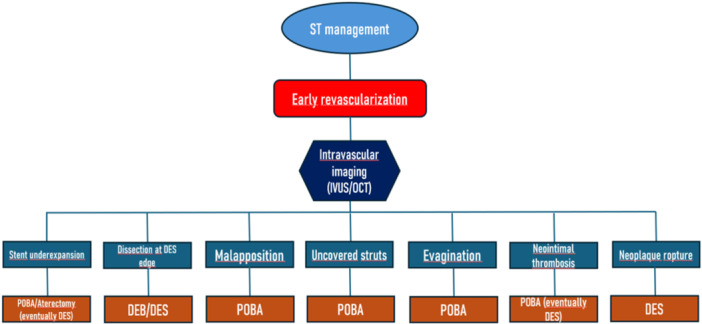
Different strategies of stent thrombosis treatment according to the underlying causes. DEB, drug‐eluting balloon; DES, drug‐eluting stent; IVUS, intravascular ultrasound; OCT, optical coherence tomography; POBA, plain old balloon angioplasty.

Once vessel perfusion has been restored, the use of intravascular imaging (IVUS or OCT) should be employed to understand the cause of ST and, therefore, to optimize the outcome, as several trials showed [64, 65, 66]. The main concept highlighted by trials is to minimize the implantation of additional DES, which should only be deployed in the event of damage to the previous stent caused by intracoronary atherectomy (the latter used in cases of ST associated with significant intrastent restenosis), dissection, plaque at the stent edge, rupture of a vulnerable neo‐atherosclerotic plaque, or neointimal thrombosis [67], as summed in Figure 2.

## 8 Preventive Strategies

In order to reduce the risk of ST, prevention must be based on four cornerstones: the correct performance of PCI (including the use of the right stent for the right lesion and optimisation of angioplasty), the indication for antithrombotic therapy, the reduction of neoatherosclerosis (with the formation of vulnerable intrastent neoplaque), and the control of the associated inflammatory storm.

### 8.1 The Role of the Different Type of Stent

The history of PCI is marked by crucial milestones; from the first balloon angioplasty performed in 1977 by Andreas Grüntzig, another milestone date is 1986, with the introduction of the BMS [67], which, however, after initial success, demonstrated a tendency to acute ST. Further advancement was represented by the introduction in 2003 of first‐generation DES, the CYPHER sirolimus and TAXUS paclitaxel‐eluting stents (PES), which, while demonstrating superiority over BMS, showed a new dark side to PCI, namely late ST and very late ST [2]. This leads to the second‐generation stents, which have demonstrated a lower tendency to thrombosis, probably due to the lower thrombogenicity of their cobalt or platinum chromium platform, their lower thickness, a more biocompatible coating, and the different anti‐proliferative drug [68]. For these reasons, stents with bioresorbable polymer (bioresorbable vascular scaffolds, BVS) and biodegradable polymer (BP‐EES) have been developed. The ABSORB BVS (Abbott Vascular, Santa Clara, CA, USA), composed of poly‐L‐lactic acid (PLLA), was the first BVS to gain widespread clinical use and substantial study in trials such as ABSORB II, III, and IV. Initial findings demonstrated promising outcomes in terms of restenosis and target lesion failure (TLF). However, longer‐term follow‐up data revealed significant limitations: in fact, the Absorb scaffold was associated with higher rates of TLF and scaffold thrombosis compared to second‐generation DES [69]. Specifically:

- At 3 years, TLF rates for Absorb were significantly higher (11.7% vs. 7.5%, *p* < 0.001);
- Scaffold thrombosis rates were also increased (1.3% vs. 0.6%, *p* < 0.001), primarily due to incomplete strut resorption and malapposition.

Recently, an important meta‐analysis evaluated the relative safety and efficacy of current marketed stents, including bioresorbable ones, including 147 studies for 126 526 patients. The results showed that all contemporary DES were superior to BMS and PES in reducing the risk of ST and MI recurrence at 1 year, but also to BVS, without a significant difference in terms of all‐cause mortality or cardiac mortality [70]. Therefore, in clinical practice, in order to reduce the risk of ST, the use of this type of stent is indicated. Of note, complete degradation of BVS is achieved in 1−4 years; thus, the benefit of BVS may emerge after 1 year after the implantation: considering that ST occurs in 1% of patients during the first year and then in 0.5% per year thereafter, the benefit, if present, would be paramount. Consequently, extended follow‐up of ongoing clinical trials would provide more insight into this device [71]. The introduction of future BVSs (thinner and at the same time more resistant, and with better resorption kinetics) will open the doors to a new era of PCI. Among the BVS technologies, the magnesium‐based scaffold Magmaris (Biotronik AG, Bulach, Svizzera) has shown encouraging results in terms of mechanical properties and safety. In BIOSOLVE‐II and BIOSOLVE‐III trials, the 12‐month cumulative incidence of TLF was 5.1%, with scaffold thrombosis rates comparable to those of second‐ generation DES [72]. Furthermore, the most recent BIOSOLVE‐IV registry.

showed that in patients treated with Magmaris, the 24‐month rate of definite or probable scaffold thrombosis was 0.8%, half of the scaffold thromboses occurred after premature discontinuation of antiplatelet/anticoagulation therapy, and only one scaffold thrombosis occurred beyond the 6‐month follow‐up, on day 391 [73].

Despite the promising features of bioresorbable magnesium scaffolds, their use is currently recommended within controlled clinical trials or registries to collect long‐term safety and efficacy data. This cautious approach aims to identify specific lesion subsets and patient profiles that can benefit most from this evolving technology.

### 8.2 PCI Optimization for ST Risk Reduction

Coronary angiography represents the gold standard for the diagnosis and treatment of ACS. However, the use of intracoronary imaging techniques is necessary to optimize the outcome of PCI, including reducing the risk of intrastent thrombosis: a large meta‐analysis of 31 studies and 17 882 patients, comparing angio‐guided versus imaging‐guided procedures, showed a significant reduction in MI (OR: 0.72; [0.52; 0.93] and repeat target vessel revascularization (OR: 0.74 [0.58; 0.90]), and ST (OR: 0.42 [0.20; 0.72]); in addition, the use of intracoronary imaging in patients with ACS reduced the incidence of ST (0.6% *vs.* 1.2%; *p* = 0.005) [74].

### 8.3 The Case of ST in Coronary Bifurcations: Challenges and Predictors

Coronary bifurcations represent a unique challenge in PCIs due to their complex anatomy, which predisposes these sites to atherosclerosis and subsequent thrombosis. Hemodynamic disturbances and unbalanced wall shear stress at bifurcation points significantly contribute to these risks. The implantation of stents in bifurcations is associated with a higher incidence of ST, with rates reported as high as 1.2% in large registries such as BIFURCAT [75].

The provisional stenting technique, which involves the placement of a single stent in the main branch with optional side‐branch treatment, has emerged as the preferred strategy in most bifurcation lesions due to its simplicity and reduced procedural risk. Data from the BIFURCAT registry highlight that the provisional technique is associated with a lower incidence of ST compared to two‐stent strategies (1.0% vs. 2.1%, *p* = 0.004). This benefit is likely attributable to reduced metal coverage and minimized mechanical disruption at the carena.

In cases where a two‐stent strategy is necessary—such as in true bifurcation lesions with significant side‐branch involvement—careful attention to stent deployment and optimization techniques is crucial. This strategy, while effective in restoring patency, increases the risk of ST due to factors such as stent overlap—and consequential high amount of exogenous material—suboptimal apposition, and incomplete lesion coverage. The use of intracoronary imaging, including IVUS or OCT, has been shown to mitigate this risk by guiding stent placement and ensuring adequate apposition and expansion. Furthermore, the adoption of the final kissing balloon technique has been associated with a significant reduction in ST risk (HR 0.48, 95% CI 0.29−0.82, *p* = 0.007), underscoring its importance in procedural optimization [75].

Future research should focus on refining stenting techniques and optimizing pharmacological strategies, particularly in patients requiring two‐stent approaches. Moreover, ensuring the adoption of imaging‐guided PCI in complex bifurcation lesions may help mitigate the risk of this potentially life‐threatening complication.

### 8.4 PCI Stentless Strategy: The Role of Drug‐Coated Balloons (DCB)

The concept of a stentless coronary revascularization strategy has gained increasing attention in recent years, particularly with the development and clinical validation of DCBs. By delivering an antiproliferative drug without leaving a permanent metallic scaffold, DCBs inherently eliminate the substrate for ST and may therefore represent a rational approach for ST prevention in selected clinical scenarios.

The strongest evidence supporting DCB use derives from small‐vessel disease, where permanent stent implantation is associated with higher rates of restenosis and thrombotic complications. Several randomized controlled trials have demonstrated the non‐inferiority of DCBs compared with contemporary DES in terms of angiographic and clinical outcomes in this setting. In the BASKET‐SMALL 2 trial, paclitaxel‐coated balloons were non‐inferior to second‐generation DES with respect to MACE at 12 months, with sustained efficacy observed at longer follow‐up [76].

Similarly, the BELLO and RESTORE SVD trials showed favorable angiographic outcomes and low rates of target lesion revascularization with DCBs compared with BMS and DES in small coronary vessels [77, 78]. Importantly, the absence of a permanent implant was associated with very low rates of device‐related thrombosis. Beyond individual trials, a large meta‐analysis including 26 randomized and real‐world studies confirmed that paclitaxel‐based strategies are associated with comparable rates of MACE and significantly reduced risks of late adverse events related to permanent metallic scaffolds [79]. Although ST is not typically a primary endpoint in these studies, reported rates of acute or late thrombotic events following DCB angioplasty are consistently low [79].

Moreover, real‐world registries have further supported the safety of DCBs for ACS, reporting favorable outcomes not only in small‐vessel disease but also in selected cases of in‐stent restenosis and complex lesion subsets [80]. These findings suggest that, in appropriately selected patients, a DCB‐only strategy may reduce long‐term thrombotic risk by avoiding delayed endothelialization, chronic inflammation, and neoatherosclerosis associated with permanent stent implantation.

Despite these promising results, the use of DCBs remains limited to specific anatomical and clinical scenarios. Careful lesion preparation, optimal balloon sizing, and strict procedural protocols are essential to ensure favorable outcomes. Ongoing studies and longer‐term follow‐up data will be crucial to better define the role of DCBs as a preventive strategy against ST in contemporary PCI practice.

### 8.5 Anti‐Thrombotic Therapy

The use of proper antithrombotic therapy is now a cornerstone of secondary prevention in the patient with ischemic heart disease; moreover, it is now well established that early discontinuation of DAPT is associated with an increased risk of ST, as mentioned earlier.

Three major studies have shown that, in patients undergoing PCI, clopidogrel discontinuation is associated with an increased risk of ST in the first 6 months, with a median time interval for a ST event of 9 days (interquartile range [IQR]: 5.5 to 22.5 days). Beyond the first 6 months, indeed, the time for an ST event was 104 days (IQR: 7.4 to 294.8 days) [81, 82, 83]. This loss of protection could explains the occurrence of LST/VLST; nevertheless, the PARIS (*Cessation of Dual Antiplatelet Treatment and Cardiac Events After PCI*) registry (*N* = 5018) found that 74% of ischemic events (including ST) occurred while patients were on DAPT [84]: roughly one‐fourth of patients undergoing stenting, in fact, may be resistant to the platelet inhibiting effects of clopidogrel, as different methods to assess clopidogrel induced antiplatelet effect (e.g., turbidometric light transmittance aggregometry and VerifyNow PRU Test) demonstrated [85]. The failure of DAPT is mainly due to genetic variation in the hepatic cytochrome CYP2C19, which is responsible for the activation of Clopidogrel from propharmaceutical to active drug [86]. In order to overcome the limitations of Clopidogrel, new P2Y12 adenosine diphosphate receptor blockers, Ticagrelor and Prasugrel, whose superiority over Clopidogrel has been respectively demonstrated in the PLATO and TRITON‐TIMI38 studies (see Table 1), as previously enunciated, have taken the field in clinical practice. In order to avoid intrastent thrombosis, especially in patients at high ischemic risk, the use of these drugs as a second antiplatelet is therefore indicated; furthermore, pre‐treatment with a loading dose, which has been shown to reduce the risk of acute ST [87, 88] in the absence of increased incidence of bleeding, is also recommended in the 2023 ESC Guidelines for the management of ACS [19].

Furthermore, after a PCI for ACS, the default duration of DAPT is 12 months, which can be shortened or extended according to the ischemic and bleeding risks of the individual patient (using tools as PRECISE‐DAPT and DAPT‐SCORE, respectively). In the SMART‐DATE (*Safety of 6‐month Duration of Dual Antiplatelet Therapy After ACS*) trial 6 and 12‐month durations of DAPT was valued: although 6‐month DAPT was noninferior for MACE, the risk of MI was increased [89]. The REDUCE‐ACS (*Short‐term Dual Anti Platelet Therapy in Patients With ACS Treated With the COMBO Dual‐therapy Stent*) trial compared 3‐ and 12‐month durations: the shortest duration was noninferior for NACE, but the rate of ST was doubled [90]. Finally, the ULTIMATE‐DAPT trial was presented at the ACC 2024 congress: Ticagrelor monotherapy following 1 month of DAPT outperforms 12‐month DAPT (aspirin and ticagrelor) post PCI for reducing clinically meaningful bleeding with no increased thrombotic risk. The placebo‐controlled trial, conducted at 58 medical centers in China, Pakistan, Italy and the UK, randomized 3400 patients who had undergone DAPT for 1 month following PCI for an ACS and had no adverse cardiovascular or bleeding events to either continuing DAPT or switching to ticagrelor and a placebo for 11 months. Results showed that the primary endpoint of clinically relevant bleeding (BARC 2, 3, or 5) at 1 year occurred in 4.6% of patients continuing DAPT and 2.1% of patients on ticagrelor monotherapy (HR, 0.45; 95% CI, 0‐30‐0.66; *p* < 0.0001). The composite primary endpoint of MACE and cerebrovascular (MACCE) events showed no significant difference between groups, with 3.7% of patients who continued DAPT and 3.6% of those taking ticagrelor monotherapy experiencing such events (HR, 0.98; 95% CI, 0.69−1.39; *p* for noninferiority < 0.0001; p for superiority =0.89). No significant interactions were seen across the 12 prespecified subgroups for clinically relevant bleeding or MACCE. Additionally, net adverse clinical events (MACCE or BARC types 1−5 bleeding) were lower in the ticagrelor monotherapy group versus DAPT group (5.7% vs. 8.2%; *p* = 0.007). The current trial suggests that DAPT duration of 1 month followed by ticagrelor monotherapy may be sufficient among patients undergoing ACS‐PCI: these are important findings and may influence clinical practice [91].

Conversely, DAPT extended beyond 12 months reduces thrombotic events compared with 12‐month regimens [92]. In a meta‐analysis of 10 randomized controlled trials (*N* = 32 135), a significant interaction between DES generation and DAPT duration on risk of ST was found. Accordingly, the benefit of prolonging DAPT over 6 months in reducing ST rates was significant in patients treated with 1G‐DES (2.4% vs. 0.6%; *p* < 0.05), but not with current 2G‐DES (0.6% vs. 0.4%; *p* = NS) [93]. In order to speed up the onset of the effects of anti‐thrombotic therapy, subcutaneously injectable antiplatelet drugs, such as zalunfiban and selatogrel, have been developed; nevertheless, their actual efficacy, demonstrated in vitro, has yet to be clinically demonstrated.

Zalunfiban, a glycoprotein IIb/IIIa receptor antagonist, represents a promising option for the early management of patients with STEMI due to its rapid onset of action and reversible effects. Preclinical studies have demonstrated effective platelet aggregation inhibition within minutes of administration. Currently, the drug is under investigation in the CELEBRATE trial, which aims to evaluate its safety and efficacy in prehospital settings [93].

Selatogrel, a P2Y12 receptor inhibitor, has been designed for practical subcutaneous administration. Preliminary studies have shown substantial platelet inhibition within 30 min of administration,

making it particularly useful in patients requiring rapid antithrombotic action, such as those presenting with acute MI. The ongoing SOS‐AMI trial (*Selatogrel Outcome Study in Suspected Acute MI*) aims to determine whether selatogrel, self‐administered subcutaneously at the onset of symptoms, can reduce the incidence of all‐cause death or non‐fatal MI in high‐risk patients with a previous history of AMI. The trial involves approximately 14 000 participants globally. Patients are trained to recognize AMI symptoms and to promptly use a pre‐filled auto‐injector to deliver selatogrel, followed by contacting emergency services. The rapid action of selatogrel is designed to provide timely platelet inhibition during the critical pre‐hospital phase, potentially limiting myocardial damage and improving clinical outcomes [94].

### 8.6 The Role of Inflammation

Several studies have tried to evaluate the impact of anti‐inflammatory therapy in the acute setting, in particular evaluating their role in the prevention of cardiovascular events. We can desume that these drugs can also have an effect on ST reduction, though the ST‐specific efficacy of these anti‐inflammatory drugs has not been established in humans. The CANTOS trial, which randomized 10,061 patients with previous MI and hsCRP ≥ 2.0 mg/L, showed that canakinumab, a monoclonal antibody targeting IL‐1β was associated with a 15% reduction in MACCEs, indicating the importance of anti‐inflammatory therapy in the management of ACS residual risks [95]. Similarly, ACS patients who have taken the IL‐6 inhibitor tocilizumab showed a reduced level of troponin and inflammatory markers in the *ASSessing the Effect of Anti‐ IL‐6 Treatment in MI* (ASSAIL‐MI) trial [96]. Another strategy for lowering the inflammatory burden was to inhibit the NLRP3 inflammasome through the use of colchicine, an anti‐rheumatic drug that can alter neutrophil function and hinder the development of the inflammasome at the macrophage level. The non‐double‐blind randomized LoDoCo (Low Dose Colchicine) trial enrolled 532 patients with CAD and investigated whether taking colchicine 0.5 mg/day could reduce the risk of cardiovascular events. The results showed that low dose colchicine could indeed lower the risk of ACS [97]. However, introducing colchicine for the secondary prevention of CAD is difficult because of the modest sample size and defects in trial design that did not involve the use of inflammatory markers as a reference for disease improvement. Recently, the *Colchicine Cardiovascular Outcomes* (COLCOT) and the Low‐Dose Colchicine 2 (LoDoCo2) trials showed a promising decline in the primary endpoints in ACS patients with previous MI [98]. Other anti‐inflammatory agents, such as MAPK inhibitor losmapimod and the recombinant P‐selectin glycoprotein ligand‐1 [99], and, recently, T‐reg cell‐based treatments ‐which are able to reverse atherosclerosis in mice [100]‐ seem to appear promising and give beneficial effects in animals ‐but still not in humans‐, and they will be a challenge for human investigation to prevent each new ACS and ST.

## 9 Conclusion

ST continues to pose significant clinical challenges despite advances in stent technology and therapeutic strategies. From a clinical perspective, several established predictors of ST reflect the mechanistic pathways discussed above. These include a prior history of ST despite adequate antiplatelet therapy, implantation of first‐generation DES, presentation with ACS—particularly STEMI or NSTE‐ACS—complex PCI procedures, diffuse coronary artery disease in patients with diabetes mellitus, and concomitant chronic kidney disease.

Effective management requires a multifaceted approach, including acute revascularization techniques, careful stent selection, and personalized antithrombotic therapy. The growing understanding of the role of inflammation and genetic variability in response to antiplatelet agents further emphasizes the need for tailored treatments. New‐generation drug‐ eluting stents, optimized PCI techniques, and emerging anti‐inflammatory therapies offer promising pathways to reduce the burden of ST. However, ongoing research and long‐term clinical trials are essential to fully understand their potential and to refine treatment algorithms. As we continue to gain insights into the underlying mechanisms, the goal of reducing both early and late ST and improving patient outcomes remains to be reached.

## Conflicts of Interest

The authors declare no conflicts of interest.

## Data Availability

The authors have nothing to report.
